# Stability, Mounting, and Measurement Considerations for High-Power *GaN MMIC* Amplifiers

**DOI:** 10.3390/s23239602

**Published:** 2023-12-04

**Authors:** Vicente González-Posadas, José Luis Jiménez-Martín, Angel Parra-Cerrada, David Espinosa Adams, Wilmar Hernandez

**Affiliations:** 1 Dpto. de Ingenieria Audiovisual y Comunicaciones, Universidad Politecnica de Madrid, Calle Nicolas Tesla, 28031 Madrid, Spain; vicente.gonzalez@upm.es (V.G.-P.); joseluis.jimenez@upm.es (J.L.J.-M.); angel.parra@upm.es (A.P.-C.); 2 Indra Sistemas, S.A., Ctra Torrejón, 28850 Madrid, Spain; despinosaa@indra.es; 3 Carrera de Ingeniera Electronica y Automatizacion, Facultad de Ingenieria y Ciencias Aplicadas, Universidad de Las Americas, Quito 170124, Ecuador

**Keywords:** monolithic microwave-integrated circuit, gallium-nitride high-power amplifier, stability analysis, measurement characterization, critical points

## Abstract

In this paper, the precise design of a high-power amplifier (HPA) is shown, along with the problems associated with the stability of “on-wafer” measurements. Here, techniques to predict possible oscillations are discussed to ensure the stability of a monolithic microwave-integrated circuit (MMIC). In addition, a deep reflection is made on the instabilities that occur when measuring both on wafer and using a mounted chip. Stability techniques are used as tools to characterize measurement results. Both a precise design and instabilities are shown through the design of a three-stage X-band HPA in gallium nitride (GaN) from the WIN Semiconductors Corp. foundry. As a result, satisfactory performance was obtained, achieving a maximum output power equal to 42 dBm and power-added efficiency of 32% at a 20 V drain bias. In addition to identifying critical points in the design or measurement of the HPA, this research shows that the stability of the amplifier can be verified through a simple analysis and that instabilities are often linked to errors in the measurement process or in the characterization of the measurement process.

## 1. Introduction

Nowadays, the Internet of Things (IoT) and wireless sensor networks (WSNs) [1] are highly in-demand technologies. Specifically, their demand cover multiple scenarios, in particular, playing an important role in solving challenges related to military technologies and medicine. In this context, radio frequency and microwave technologies play a fundamental role because of the capacity they provide for the wireless transmission of detection data and for the wireless transfer of energy [2,3]. Therefore, the constant development and growth of wireless sensor networks are significantly supported by advances in radio frequency (RF) technologies [4].

WSNs have attracted attention due to their versatility and uses in various sectors, such as health care, military applications, industrial automation, and urban intelligence [1,2,5]. However, the increasing demand for mobile communication services has brought with it a critical shortage in terms of available space on the RF spectrum. Therefore, it has become necessary to move to the use of technologies such as gallium nitride (GaN) in the design and construction of antennas and sensors. However, the correct functioning of these technologies depends, to a large extent, on some of the most critical devices: high-power amplifiers (HPAs). In essence, WSNs, the best use of the IoT, and the efficient use of the RF spectrum depend on the correct design and operation of HPAs.

Nevertheless, the design of integrated power amplifiers always requires a compromise between stability and performance. Specifically, for an amplifier to be stable (that is, not oscillate), gain, power, and performance are sacrificed. Therefore, it is necessary to evaluate how much gain and power-added efficiency (PAE) should be reduced to ensure stability. There are excellent examples of applications and commercial chips where yields or powers have been prioritized [6], for example, Qorvo’s QPA3069 [7]. But in the vast majority of monolithic microwave-integrated circuits (MMICs), many of the concepts associated with the stability of on-wafer measurement or PCB (printed circuit board) mount measurement are ignored. The truth is that in the best of cases, basic concepts and knowledge are used, such as the stability factor. In [8], the problem of the stabilization of a circuit in a PCB was analyzed. Some previous works that form part of the fundamental motivation of this paper are discussed below.

In [9,10], the authors realized that using the basic concepts of linear stability [11,12] to design amplifiers did not produce satisfactory results, and despite ensuring that stability requirements were met, some of the designed HPAs oscillated. Then, the normalized determinant function (NDF) was introduced, and the design was made stable. Additionally, an explanation for the oscillations was given.

In our opinion, the NDF is a modern version of the return ratio (RR). Furthermore, it has successfully been used to design oscillators [13,14], showing its agreement with Bode’s RR. However, the NDF criterion is difficult to apply and requires that the software be prepared for its use. The main drawbacks of the NDF is that it requires access to all the internal nodes of the transistor model and that it is a small signal analysis. The above means that it is a non-viable method to perform large signal analysis, which is necessary in HPA analysis.

Delving into the line of establishing methodologies that enable a more precise analysis of stability, especially in the design of HPAs, it is worth highlighting the studies carried out in [15,16,17]. In these papers, developments leading to techniques with greater or lesser success and allowing for the analysis of the stability of MMIC designs were presented.

Although there are many approaches to the stability problem, we believe that the most appropriate and simple tool for the analysis of stability was provided in [18,19,20,21]. This is due to the identification of pole zero. The authors of [18,19,21] marketed a tool for the aforementioned analysis called STAN [22]. However, despite what has been said above, sometimes, the expert in charge of the designs encounters some undesirable surprises. For example, the MMIC can oscillate when measurements are conducted on a wafer or on a mounted chip.

In this paper, the design of an X-band HPA with 32% efficiency and an output power equal to 42 dBm and $V_{D}$ = 20 V is presented. Here, the stages of design and measurement are described, as well as the instabilities that arise and that are not design problems but problems associated with the measurement or assembly process. The most important contribution of this work is that the problems that arose in the measurements were identified and analyzed. These problems produced instabilities that generated oscillations that cannot be detected using classical stability analysis methods [12]. The design proposed in this research uses pole-zero identification [19] to identify and solve stability problems both in the design phase and in the measurement phase of the HPA-MMIC.

The rest of this paper is organized as follows. Section 2 presents a brief description of HPA technology and the design process. Section 3 describes the process of analysis of the total stability of the designed MMIC and the setup carried out for its analysis. In Section 4, the problems found in the measurements performed “on wafer” and with the device mounted on a carrier are shown. Section 5 shows the measurements carried out on the device after the reason for the instability was detected and identified and after the HPA was stabilized to make its measurement possible. Section 6 is aimed at presenting the discussion of the results. Finally, the conclusions are given in Section 7.

## 2. Issues with X HPA Design

### 2.1. Process NP15-00

WIN Semiconductors [23] has extensive experience in manufacturing HBT and HEMT MMICs of GaAs and GaN. WIN’s technology roadmap covers more than 20 processes that are available and ready for production. Furthermore, WIN is familiar with the latest developments in GaN and GaAs technology and provides continuous developments to meet requirements. In this research, we chose NP15-00 because it has a very good ability to operate satisfactorily at high frequencies. In addition, it is optimized for applications up to 40 GHz. Likewise, thanks to the fact that NP15-00 has a lower capacity than a 0.25 μm process and because it has a high power density, it is suitable for working at high power (3.5 W/mm), with high PAE and high linearity. Additionally, the NP15-00 substrate has a very good thermal dissipation capacity. GaN HEMT models contain an internal thermal subcircuit consisting of an RC circuit.

Regarding the 0.25 μm process, we would like to mention that it has a higher power density but does not achieve as high a gain as the 0.15 μm process. Therefore, we decided to work with the 0.15 μm process because this process has the great advantage of being able to provide greater gain with slightly lower power. The above has a positive impact, enabling an HPA with adequate gain for electronic warfare or RADAR applications in the X band to be obtained, with a smaller number of stages.

To sum up, the NP15-00 process is an 0.15 μm GaN on silicon carbide (SiC) technology from WIN Semiconductors [23]. This process is suitable for applications up to 40 GHz, with a cutting-edge figure of merits in terms of power density, gain, efficiency, and reliability. The typical output power density of the proposed design is 3.5 W/mm at a 20 V drain bias voltage. The MMIC process includes precision TaN resistors, high-value TiWSi resistors, MIM capacitors, inductors, air bridges via holes through the substrate, and two metal layers for interconnection.

### 2.2. Transistor Selection and Architecture of the HPA

Our goal was to design an HPA that could deliver a 6–12 GHz bandwidth, an output power greater than 42 dBm (15 W), more than 30% PAE, and an input power of less than 17 dBm, all on a chip with dimensions of 5 × 4 mm^2^. Thus, the choice of active devices and the selection of their static operating point directly determine the performance of the amplifier circuit.

In this work, the process design kits (PDKs) of the WIN NP15-00 foundry with HEMT GaN transistors were used to carry out the circuit stability simulations and maximize the power and gain of the HPA. Several WIN transistors of different sizes (e.g., 8 × 150 μm, 8 × 100 μm, 6 × 100 μm, etc.) were characterized using PDKs. AWR version 16 and ADS 2023 software were used to simulate HEMT devices with different gate widths and numbers of fingers, as well as to generalize the performed analyses as a demonstration of the degree of maturity of the PDKs provided by the WIN foundry.

**Figure 1:**
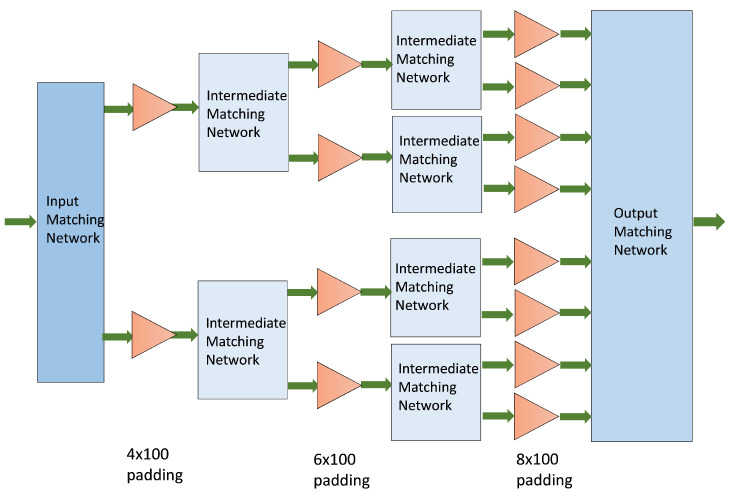
HPA architecture. Each triangle represents a transistor with padding, and the green arrows indicate the direction of the signal.

Generally speaking, the gain of the transistor decreases as the frequency increases, as well as with an increase in the size of the device. At the time of design, the largest available transistor size was 8 × 125 μm. Therefore, later versions had to be modified to 8 × 100 μm, which still offers good X-band performance and a maximum drain bias voltage of $V_{D}$ = 20 V. At present, the process provides the possibility of feeding at $V_{D}$ = 28 V, which increases the output power ($P_{out}$) of the device. Figure 1 shows the selected HPA architecture.

In the architecture shown in Figure 1, 4 × 100 μm, 6 × 100 μm, and 8 × 100 μm transistors were chosen to meet the necessary requirements. However, in the end, all the transistors that were used were 8 × 100 μm to improve the robustness of the design. For simplicity, the greatest symmetry was sought in the design of the HPA [24]. Therefore, all transistors had padding.

The transistor discussed in Section 2.1 needs to be biased at a particular operating point for operation as an amplifier. Selecting a proper bias point is a major aspect in power amplifier (PA) design. Not only does the DC bias network determine the PAE, but it also defines the PA performance over temperature. In this research, we chose to bias an AB amplifier point in both stages. The DC bias current in the output transistors (i.e., the output stage) was 241 mA × 2, 125 mA × 2 in the intermediate transistors (i.e., the intermediate stage), and 46 mA × 2 in the input transistors (i.e., input stage). Gate voltages ($V_{G}$) were the same for $V_{G1}$ (input stage), $V_{G2}$ (intermediate stage), and $V_{G3}$ (output stage) and set to −1.6 V. The gate current was approximately equal to 0 mA. It is recommended that gate currents be measured during amplifier operation. In addition, it is desirable to test $V_{G1}$, $V_{G2}$, and $V_{G3}$ current consumption separately.

### 2.3. Load Pull

Load-pull analysis can be said to be the heart of PA design. This is the most important step in the design of a high-efficiency PA and should be carried out with care. The load-pull technique determines the optimum value of the load impedance that should be provided to the amplifier’s output to achieve maximum PAE, optimum delivered power, and high gain and linearity. The load reflection coefficient ($\rho_{L}$) is given by (1).

(1) $$\rho_{L}=\frac{Z_{L}-Z_{out}}{Z_{L}+Z_{out}}$$

where $Z_{L}$ is the load impedance and $Z_{out}$ is the output impedance. $Z_{out}$ is fixed and cannot be altered. Thus, it is evident that the load reflection coefficient ($\rho_{L}$) becomes a function of $Z_{L}$. Any variation in the load impedance would cause the load reflection coefficient to vary, which is the basic philosophy behind the load-pull technique. Basically, the simulator (i.e., the ADS simulator or the AWR simulator) varies the load impedance over a selected area on the Smith chart and plots contours with various values of PAE and $P_{del}$. The optimum point is determined by the intersection of the ${\mathrm{PAE}}_{max}$ and $P_{del,max}$ contours. In cases with no intersection, the most proximal point is chosen as the optimum impedance point

The input tuner is first adjusted to deliver maximum power from the signal source to the input of the active device, after which the output tuner is adjusted to deliver maximum output power to the load. As important points when characterizing load pull, the following are taken into account:

- Once the adjustments are completed, the impedance tuners are disassembled, and the desired load ($\rho_{L,opt}$) and source reflection coefficients ($\rho_{S}$) are obtained by measuring the impedances of the impedance tuners;
- The spectrum analyzer and the power meter connected to the output impedance tuner make it possible to measure the exact value of the output power while simultaneously observing the output spectrum in the spectrum analyzer;
- In case of lower harmonics, the optimum load impedances can be obtained easily; however, a problem does occur when the harmonic impedances are considered;
- In general, the maximum output power is determined from the load impedance of the fundamental frequency, but the efficiency varies according to the harmonic load impedance.

**Figure 2:**
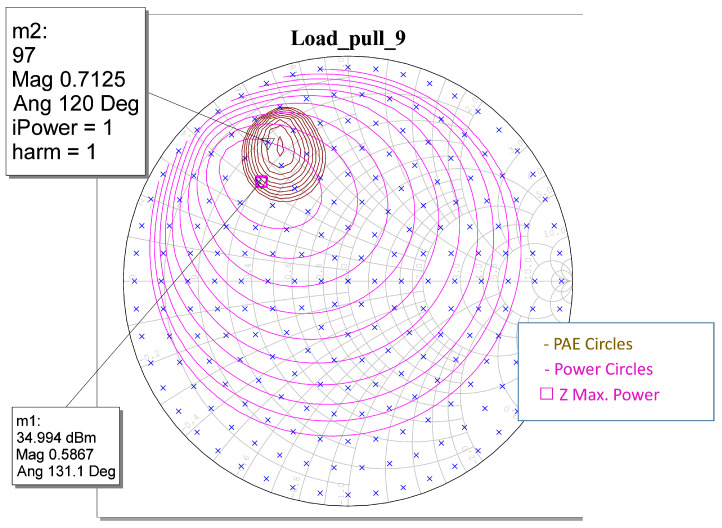
Load-pull results of 8 × 100 μm transistors at 9 GHz. Brown circles represent PAE, and purple circles represent gain.

**Figure 3:**
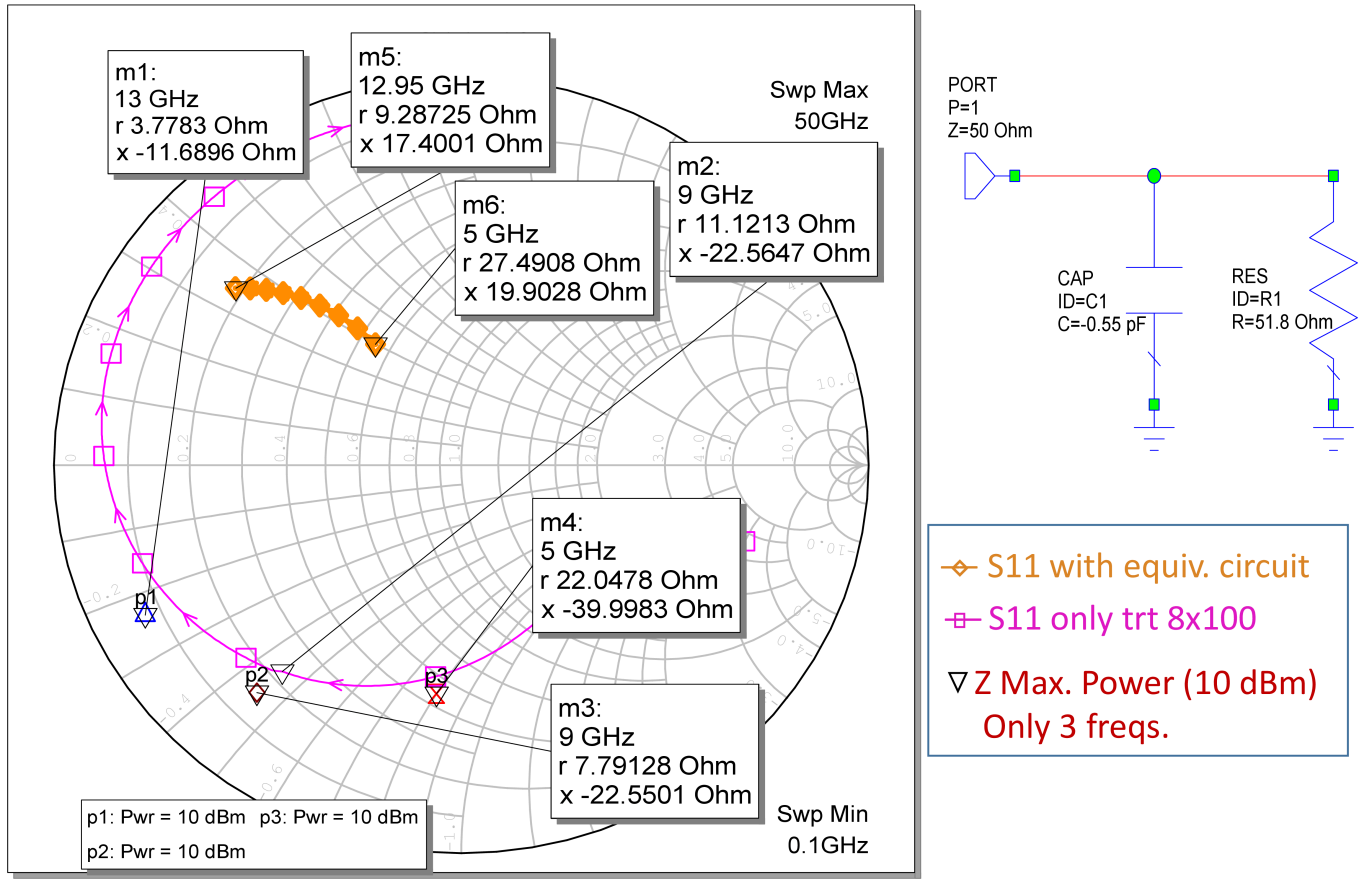
Equivalent circuit of load 8 × 100 μm transistors.

In this research, the WIN PDK was used to determine loading under the optimum fundamental and harmonic loading conditions. For example, Figure 2 shows the results obtained for the load-pull analysis with an AWR of 8 × 100 μm transistors at a 9 GHz frequency. In addition, the large signal models for the PDK transistors were verified, and the optimum load impedance of the HPA transistor was extracted. With this impedance, an equivalent input circuit was extracted, normally *RC*, where *C* is negative to simulate the non-Foster behavior of the optimum impedance. This equivalent circuit is used to construct the matching networks depending on the chosen architecture. Figure 3 shows a comparison of the measurement results of the impedances of the load pull and the equivalent circuit extracted for the 8 × 100 μm transistor. In the same way, we proceeded with the other 4 × 100 μm and 6 × 100 μm transistors, as well as transistors of others sizes.

### 2.4. Matching Network Strategy and Design

This is perhaps the most critical point of the design. The bias network is part of the matching network, and even at the input, it can help stabilize the amplifier. The input and output matching networks are responsible for realizing the optimum impedances, which are determined by load-pull data collected at gate and drain of the active device, respectively. In addition to optimum impedances, the design needs to include low-loss matching elements to avoid the loss of power at the output or input stage. From our point of view, there is no single methodology for the design of MMIC matching networks. However, we aim to establish the necessary knowledge and clear guidelines that each designer must interpret and use for the design of these networks.

It is important for the design strategy of the adaptation networks to assume that all input and output networks of the different transistors and amplification stages are basically composed of transmission lines (TLs); capacitors; and, in some cases, coils (L) at low frequencies and TLs at higher frequencies.

The gain–bandwidth limitation to impedance, known as the Bode–Fano criterion [25], for an *RC*-parallel element is given by (2).

(2) $${\int_{0}}^{\infty }\ln\left|\frac{1}{\Gamma (\omega )}\right|d\omega \leq \frac{\pi }{RC}$$

**Figure 4:**
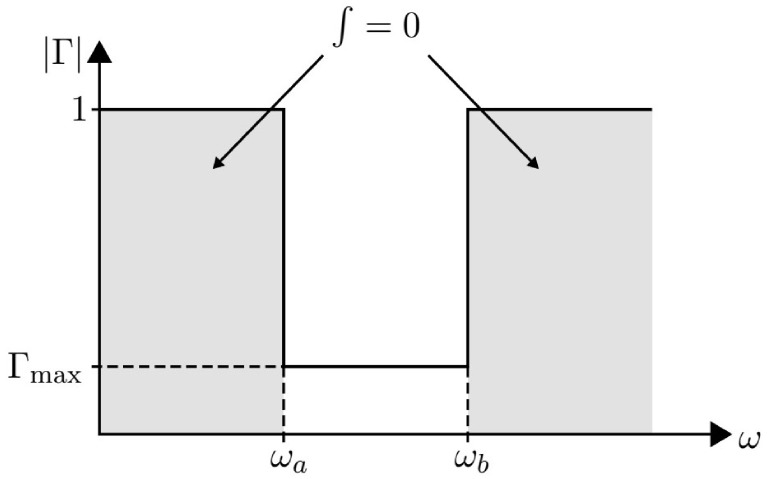
Response of an ideal adaptation network.

The goal of a wideband adaptation network is to achieve a Γ value as small as possible within a bandwidth specified by $\omega_{b}-\omega_{a}$. Therefore, the contribution of the integral when outside the $\omega_{b}-\omega_{a}$ bandwidth should ideally be zero. The response of an ideal adaptation network is shown in Figure 4.

The response of the adaptation network is indistinguishable from that of an ideal filter. The reflection coefficient of an ideal filter should be 1 outside the band and should remain below a maximum value ($\Gamma_{max}$) inside the band. Furthermore, assuming that Γ = constant = $\Gamma_{max}$ within the band, the equation can be expressed as (3).

(3) $${\int_{\omega_{a}}}^{\omega_{b}}\ln\frac{1}{\Gamma_{max}}d\omega$$

There, solving (3), we obtain (4).

(4) $$\ln\frac{1}{\Gamma_{max}}\left(\omega_{b}-\omega_{a}\right)$$

Next, we apply inequality (2) and obtain (5) and (6), where ω=2πf.

(5) $$\ln\frac{1}{\Gamma_{max}}\leq \frac{\pi }{\left(\omega_{b}-\omega_{a}\right)RC}$$

(6) $$\ln\frac{1}{\Gamma_{max}}\leq \frac{1}{2\left(f_{b}-f_{a}\right)RC}=\frac{1}{2\Delta fRC}$$

Then, solving for the parameters of interest in (6), we obtain (7) and (8).

(7) $$\Gamma_{max}\geq e^{-\frac{1}{2\Delta fRC}}$$

(8) $$\Delta f\leq -\frac{1}{2\ln\Gamma_{max}RC}$$

In addition, the Bode–Fano criterion expressed in terms of the load quality factor is given by (9).

(9) $$\begin{array}{c} \Gamma_{max}\geq e^{-\pi \frac{Q_{loaded}}{Q_{ofload}}}=e^{-\pi \frac{Q_{2}}{Q_{1}}} \end{array}$$

where $Q_{ofload}$($Q_{1}$), i.e., network *Q*, is given by (10), and $Q_{loaded}$ ($Q_{2}$), i.e., bandwidth *Q*, is given by (11).

(10) $$\begin{array}{c} Q_{ofload}=Q_{1}=R\omega_{o}C=\frac{R}{X_{C}} \end{array}$$

where $X_{C}$ is the absolute value of the load reactance, and *R* is the load resistance.

(11) $$Q_{loaded}=Q_{2}=\frac{f_{o}}{\Delta f}$$

where $f_{o}$ is the center frequency. Furthermore, $Q_{loaded}$ is upper-bounded (see (12)).

(12) $$\begin{array}{c} Q_{loaded}\geq -\frac{Q_{ofload}\ln\Gamma_{max}}{\pi } \end{array}$$

The Bode–Fano criterion provides an absolute limit on bandwidth for a given device (*Q*) and an achievable return loss. This implies that we need impedances grouped according to frequency and with the lowest *Q* possible. This is translated, at a practical level, as a sacrifice in the gain of the device to make it more adaptable in such a way that the impedances are the closest to each other with a low *Q*. This also improves the stability. This grouping is usually performed using the padding. The above implies limitations, in terms of limits, on the adaptation of the input and output of the active devices. And these limits can be approximately calculated with the help of the equivalent circuits to which we apply equivalent input impedance and, above all, the output of the device. This is the most critical part of the two networks to be realized.

**Figure 5:**
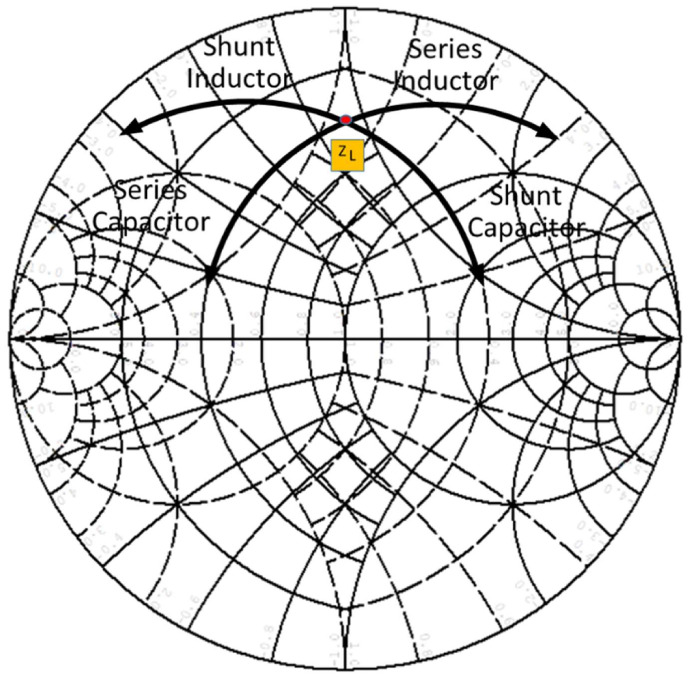
Displacement from an impedance ($Z_{L}$).

One determining factor in the design of adaptive networks is the knowledge of the Smith chart and the adaptation of impedances by using it. It is important, as Figure 5 shows, to know how we move each time we add a matching element, i.e., L(*TL*) or C. Figure 5 show the displacement from an impedance ($Z_{L}$) of the load when an L or C is added to it, either serially or in parallel, always attempting to obtain the lowest displacement, which translates into higher bandwidth.

**Figure 6:**
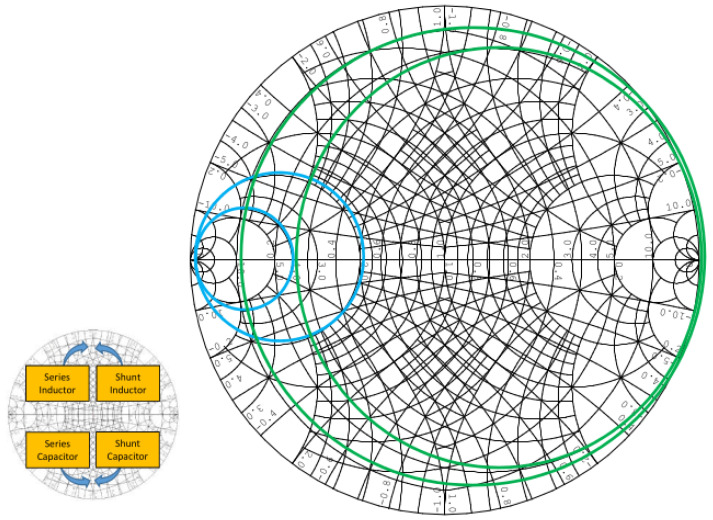
Circles of constant R (green circles) and constant G (blue circles).

Another important factor when selecting jumps or elements is to take into account the space limitations of the design, especially when adding an L component synthesized with TLs. The limitations on both bandwidth and space mean that jumps in the values of L and C lead to a lack of a clear methodology to follow for the selection of the aforementioned values. Most of the time, it is the experience and knowledge of the designer himself that sets the standards in the jumps. Sometimes, the designer looks at the movement within the circles with constant real parts, and it is that movement (see Figure 6) that largely determines which jump is selected.

In Figure 6, the green circles represent circles with constant real part (R) in the impedance (Z). In this case, we move within an impedance Z = R + jX with R constant, where R = ReZ is the resistance and X = ImZ is the reactance. Additionally, blue circles represent Y = G + jB admittance (Y) circles, where G = ReY is the conductance and B = ImY is the susceptance. Therefore, in this case, we would move with a constant conductance (i.e., constant G). Figure 6 tells us the following: (1) when we put a series impedance (L or C) then we move inside the green circles, and (2) when we put a parallel impedance (L or C) then we move inside the blue circles. Additionally, as is normally done in this type of design, we have superimposed the Smith charts of Z and Y.

Another important factor is to know the impedance you want to match, as it has complex characteristics, provided that equivalent circuitry is available. In this case, there are two possibilities:

1. Absorb the reactive part in the matching network (higher-bandwidth solution);
2. Resonate the reactive part (lower-bandwidth solution).

**Figure 7:**
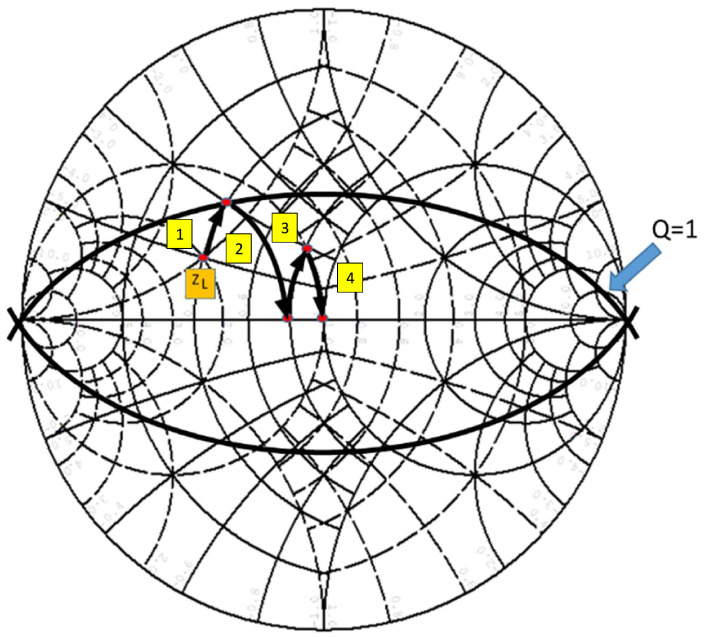
Example of four-step matching. The first red dot represents $Z_{l}$ (the impedance that we want to adapt). The second red dot, signaled with step 1 (yellow), represents the impedance value with the first adaptation element. Then, the third red dot, signaled with step 2 (yellow), represents the impedance value with the second adaptation element. Thus, successively, we reach the last red point, signaled with step 4 (yellow). This point represents the final impedance ($Z_{o}$) resulting from the desired adaptation. Likewise, Q = 1 is the adaptation quality factor.

In practice, when broadband performance is needed, it is customary to use multiple (i.e., more than three) element designs. The advantage of these designs is that they can determine the overall quality factor (*Q*) of the network. The lower the *Q*, the more broadband the design. The Smith chart is a valuable tool in this case as well. The Q of a series impedance circuit is the ratio of the reactance to the series resistance. Each point on the Smith chart therefore has a *Q* associated with it. The locus of impedances on the chart with equal Q values is a circular arc that passes through the open-circuit (O/C) and short-circuit (S/C) load points. Q circles are shown in Figure 7.

Constant *Q* circles can be used to provide limits within which the matching network should remain to provide the required operational bandwidth. The design process, as illustrated in Figure 7, is as follows:

1. Normalize the source and load impedances with a convenient value ($Z_{o}$) and plot them on the Smith chart;
2. Plot the *Q* circle that corresponds to the required overall quality factor (a low value less than 3 is normally selected);
3. If the matching network is a π network (e.g., the last element is a parallel component), move away from the load along a constant g circle on the immittance chart until the *Q* circle is reached. If the matching network is a T network (e.g., the last element is a series component), move away from the load along a constant r circle until the *Q* circle is reached;
4. Introduce an element with a sign opposite to that of the first one until the real axis is reached;
5. Repeat the previous process, introducing as many elements as required until the source is reached.

As an example of using the circles in Figure 6 and using the movements in Figure 5, Figure 7 shows how the impedance $Z_{L}$ (the first red dot), which is the impedance that we want to adapt, is adapted to $Z_{o}$ (the last red dot), which is the final impedance resulting from the desired adaptation. To do this, in step 1) (explained above) we introduce a series L_1_ and move inside the green circle, whose real part of the load impedance R_1_, to the second red dot Z_1_ = R_1_ + jX_1_. This point will have an admittance Y_1_ = 1/Z_1_ = G_1_ + jB_1_. Next, we introduce a parallel capacitor, C_1_, and move within a blue circle to the third red dot. At this point, we will have an admittance Y_2_ = G_2_ + jB_2_. Additionally, this point will have an impedance Z_2_ = 1/Y_2_ = R_2_ + jX_2_. Again, we place a series inductance to move within the green circle until the fourth red dot. At this point, we will have the impedance Z_3_ = R_3_ + jX_3_. Therefore, we calculate the admittance value Y_3_ = 1/Z_3_ = G_3_ +jB_3_, and proceed to the last step. In the last step, we place a parallel capacitor and move within a blue circle of constant conductance, to the center of the Smith chart. This is the last red dot, and represents both Y_4_ = Y${}_{o}$ and Z_4_ = 1/Y_4_ = Z${}_{o}$.

It is important to highlight practical aspects such as the fact that the bias network is usually integrated with one of the elements used in the matching, usually the first parallel inductor closest to the transistors, although there is nothing written about this and it depends on the designer. The routes in the match can be different, and it is not necessary to use them, although it seems logical to do so, since the physical implementation is what limits the choice of network. In summary, it is important to remember the following aspects:

- The more elements, the greater the bandwidth but also greater the losses. It is not usually practical for networks to have more than three or four poles (the design determines the number of poles and the used architecture).
- Attempt to integrate the reactive part into the adaptation network; if not, it is then possible to resonate it partially (preferably) or completely.
- Integrate polarization networks into adaptation networks.
- Attempt to use interspersed low-pass and high-pass sections.
- Make intensive use of the Smith chart, looking for short paths.
- Given the above, obtain an initial solution and proceed to refine it through optimization.
- Keep in mind that the limitations on space and values mean that the jumps are not selected according to a clear methodology but the designer’s own knowledge and experience.

In [26,27,28], some techniques for the design of MMIC networks were developed which using different procedures to adapt impedances in monolithic circuits of all types. However, from our point of view, in most cases, these techniques are useful only for the specific problem explored in the articles in which they were presented and are difficult to apply to the design of MMIC networks in a systemic way.

Nevertheless, it is worth mentioning the work of Niclas, especially that presented in [29], where resistive equalization methods for the realization of PA were established, which, in most cases, are used intuitively by designers in both classical and distributed topologies without knowing it.

One last comment on the design of power amplifiers is that it is advisable to design matching networks starting from the output network and finishing at the input network. Additionally, always look for the best possible symmetry in the design process.

### 2.5. Simulation Setup and Strategy

**Figure 8:**
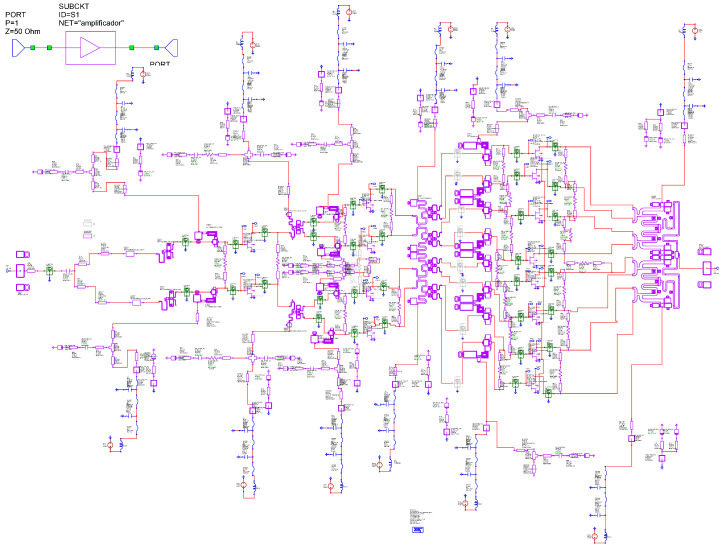
Setup of the test-bench *S* parameters.

**Figure 9:**
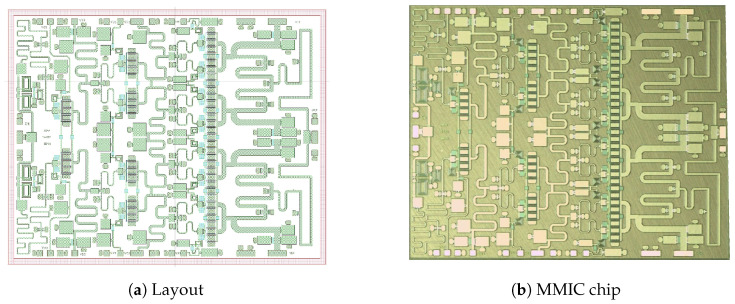
Layout of the MMIC and the chip.

**Figure 10:**
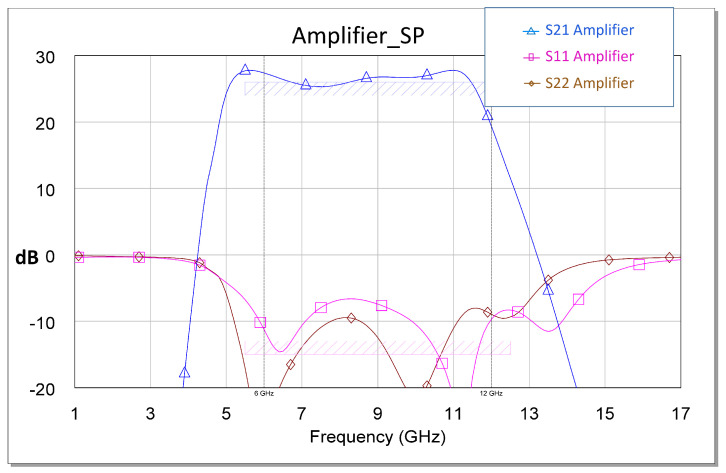
*S* parameters obtained through EM simulation.

This section describes the setup of the most important measurements and performed simulations. We placed the test bench for measurements of the total-circuit *S*) parameters as shown in Figure 8. The circuit diagram shown in this figure corresponds to Figure 9. In addition, all possible lines, paths, and components (L and C) were analyzed using electromagnetic (EM) simulation, except for the transistors. Once the circuit was optimized, we proceeded to carry out the simulations. Figure 10 shows the *S* parameters. Since priority was given to the EM response of the networks, all graphs of the responses were obtained through EM simulations.

In the first phase, optimization was carried out at the circuit level, where the lines and passive elements were simulated with the models provided by the foundry. Next, electromagnetic simulation was carried out, where the lines and other passive elements were electromagnetically simulated. In the first phase, a response close to or slightly better than the desired one was obtained. Then, when passing the circuit to the EM model, simple optimization was carried out. The PDK model and the working frequencies make the circuit response close to the EM response. Another notable aspect is that EM simulation is usually a computationally intensive and time-consuming process.

It is important to mention that to obtain the circuits from both the circuit-based and EM simulations, analysis of the linear response (small signal and measurements of the *S* parameters) was first carried out; then, as the main objective of the HPA, the response to large signals (non-linear measurements ($P_{out}$, i.e., output power), PAE (power-added efficiency), and $G_{P}$ (power gain)) was analyzed. All large-signal characterization was performed both in the frequency domain and taking into account the input excitation power ($P_{in}$) of the circuit.

Taking all these observations into account, the impedance adaptation and transformation networks making up the final amplifier were optimized as a design strategy for the total circuit, always starting from the output and ending at the input network.

**Figure 11:**
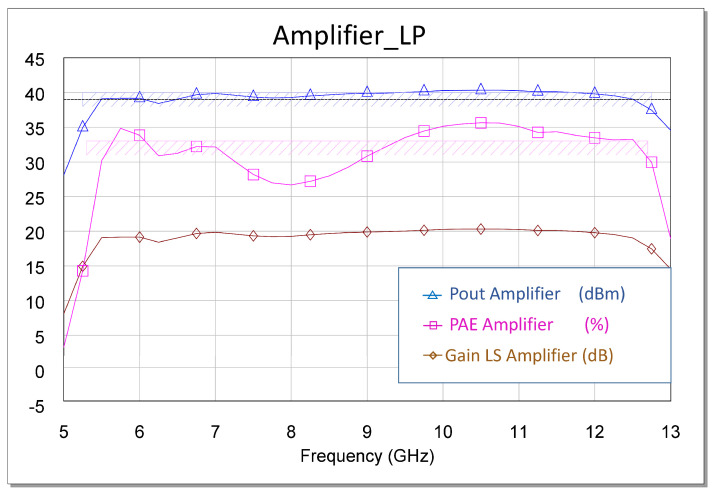
Optimized non-linear EM simulation results of the amplifier with $P_{in}$ = 16 dBm.

Another fundamental observation is that this strategy was refined in the final design stage by performing a final optimization of the total circuit with the EM model. Based on experience, the above is due to the fact that the real response of the built circuit is similar to that of the electromagnetically simulated circuit. Figure 11 shows the expected non-linear power and performance results.

**Figure 12:**
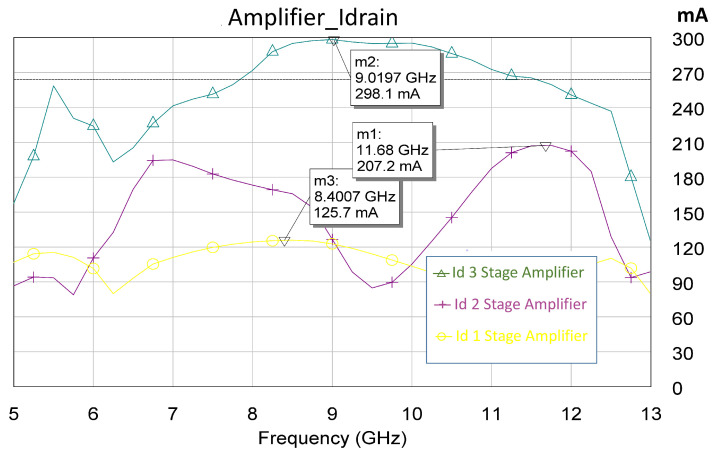
Values of the drain current in each of the stages in the presence of an excitation signal ($P_{in}$ = 16 dBm). The dashed black line is the average value of the third stage current. This value serves as a guide.

Another fundamental analysis consists of observing the polarization behavior in the presence or absence of a signal, as shown in Figure 12.

## 3. Stability of the Design

For the HPA design, the basic principle is that it is stable and not self-excited and has an amplification effect. It combines the stability design and the bias structure design. The stability analysis of the circuit is divided into four steps. The first two are related to the design itself.

In the first step, the stability of the devices is ensured by adding an *RC* padding at the input of the power devices, because the solution involves the artificial introduction of lossy elements, which changes the negative resistance at the device port to a positive value. We combined stabilization networks and biasing circuits to realize a compact design. The introduction of a lossy element into the padding involves the establishment of a compromise between the device gain and stability. Additionally, this provides input and output impedances with adaptation or transforming networks that can be synthesized more easily and with a greater bandwidth.

The values of the circuit were also optimized considering the following general rules:

1. Minimum disturbance of HPA performance;
2. A sufficient stability margin to ensure stable performance, considering the technological dispersion of the circuit parameters;
3. A sufficient gain margin to ensure the gain of the X HPA MMIC;
4. A sufficient PAE and $P_{out}$ to ensure HPA performance;
5. A minimal chip area.

In the second step, parallel resistors are introduced between the transistors, the purpose of which is to stabilize the amplifier in the odd modes of operation that may arise, as also studied by Platzer [9]. These resistors do not suppose any additional loss a priori; therefore, their dimensioning in power is imposed by the asymmetries of the total circuit.

The other two steps are related to the circuit analysis, where instabilities are analyzed at a nominal operating point for both small and large signals. The evolution of the steady-state oscillatory solution is studied in comparison with the key design parameters determined using harmonic balance.

### 3.1. Small-Signal Stability Analysis

Platzer et al. [9] reported the notable drawback that when they designed MMIC HPAs, the amplifiers almost always oscillated, although they used the stability conditions proposed by Rollet [30] and their updated expressions [12]. This, as the author himself recognizes, was the result of the fact that these stability factors could not detect the internal instabilities of the MMIC normally associated with the appearance of even modes.

Furthermore, it is necessary to highlight the distinction between instabilities because from the point of view of mathematical analysis, these can be seen as the solution of the differential equation that characterizes the response of the device. When there is no signal at the input of the device or the signal has a small value, the bias point of the MMIC is not altered by the presence of the signal. At a practical level, the stability of a direct current (DC bias) or a small signal is analyzed. Classical methods only detect small signal instabilities. When the presence of a signal immediately modifies the MMIC bias, we obtain a solution of large-signal stability.

In the case in which an HPA-MMIC oscillates in the absence of an input signal (that is, it is only polarized), this oscillation is considered to be associated with small signal instability. In addition, this oscillation or instability cannot be detected using classical methods [30]. As Platzer et al. [9] attest, to a large extent, this oscillation is the result of the architecture used for the design of these HPA-MMICs. Additionally, in this case, the stability factor (K) [30] is not a good predictor of the internal instabilities of the MMIC because it does not detect the appearance of the poles associated with the oscillation. Furthermore, it should be noted that the stability analysis of a small signal amplifier is carried out by linearizing the circuit equations about its DC point. Faced with these problems, Platzer et al. [9] proposed the use of the normalized determinant function (NDF) as an appropriate technique to detect the instabilities mentioned above. In this way, the authors obtained a tool that accurately detected instabilities and justified the oscillations that appeared. On the other hand, the main drawback of this technique is the fact that it is necessary to use the equivalent circuits of the transistors to calculate the NDF. Moreover, it becomes extremely complicated when carrying out large signal analysis.

We propose the use of the pole-zero identification technique [19] for the analysis of HPA-MMIC instabilities. This is because this technique is based on the fact that for a given system, any closed-loop transfer function shares the same denominator, with poles that are the same as the roots of the characteristic equation of the system. For the system to be stable, all its poles must be located in the left half-plane of the complex plane. Therefore, the appearance of complex conjugate poles on the right half-plane is associated with oscillations. Finally, the circuit can be stable for a given set of values of its parameters, including its components and load impedance.

Stability analysis is based on the calculation of a closed-loop transfer function of the multifunction circuit to which the pole-zero identification is applied [18,19,21]. It should be noted that all possible closed-loop transfer functions defined on a circuit line share the same denominator and therefore contain the same number of poles. On the contrary, the zeros depend on the particular definition of the transfer function.

**Figure 13:**
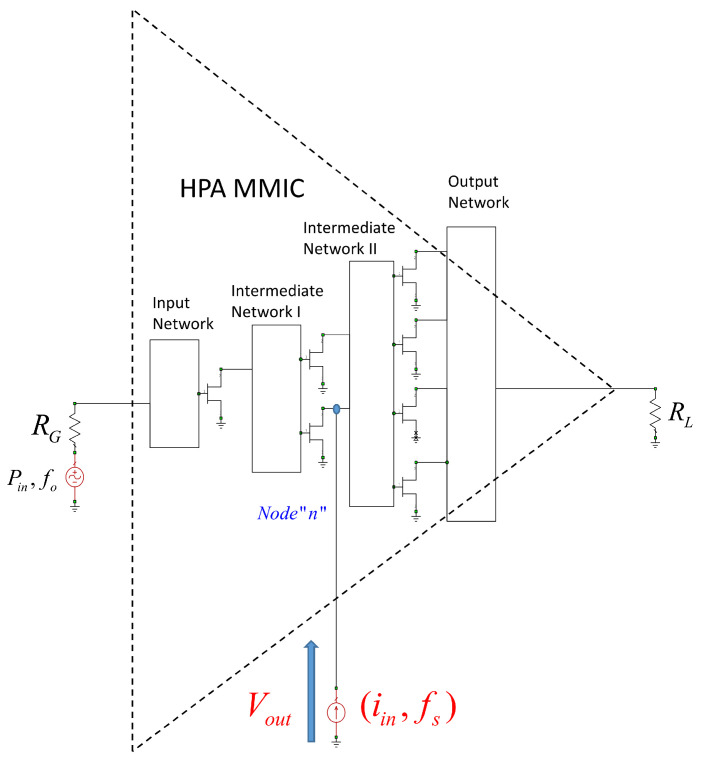
Scheme of the analysis of Z(f) using pole-zero identification.

Identification can be performed by evaluating either the Z(f) function or its inverse function, i.e., Y(f). These are functions that are extracted by inserting a small signal generator into the circuit, as shown in Figure 13. Reading the value of $\frac{V_{out}}{\left(i_{in},f_{s}\right)}$, we would obtain Z(f) and, therefore, the poles and zeros of the designed MMIC. The position is the result of the designers’ experience [21], although normally in HPA-MMIC topologies, the gates and drains are the most-often-recommended places to evaluate the presence of unstable poles. Figure 13 shows how the generator is placed in a node in a drain of one of the transistors.

**Figure 14:**
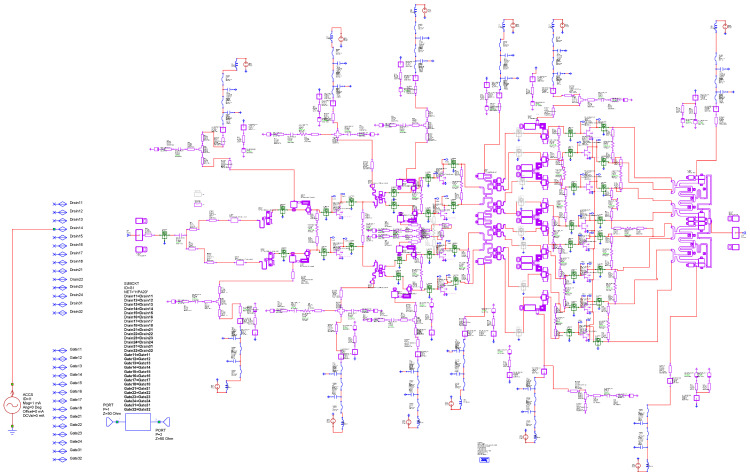
Setup of the small-signal stability analysis.

Figure 14 shows the measurement setup implemented in AWR for the X HPA. This figure represents the complexity of the actual implementation of Figure 13 in the realized design. Figure 13 shows where a node would be located in a simplified way for an architecture with four output transistors. The design studied and carried out in this work has transistors in the output stage. Here, Z(f) is analyzed and studied, both in the gates and in the drains of each of the transistors that make up the MMIC.

For the calculation of this function, a small-signal current generator at a frequency of *f* is introduced in parallel at a given circuit node, obtaining the ratio (*Z*(*f*)) between the node voltage and the introduced current. The generator frequency (*f*) is swept, and zero-pole identification is applied to *Z*(*f*). Since the zeros depend on the particular definition of the transfer function, pole-zero cancellations may occur at particular locations of the current source. Therefore, different observation nodes must be considered for this analysis.

**Figure 15:**
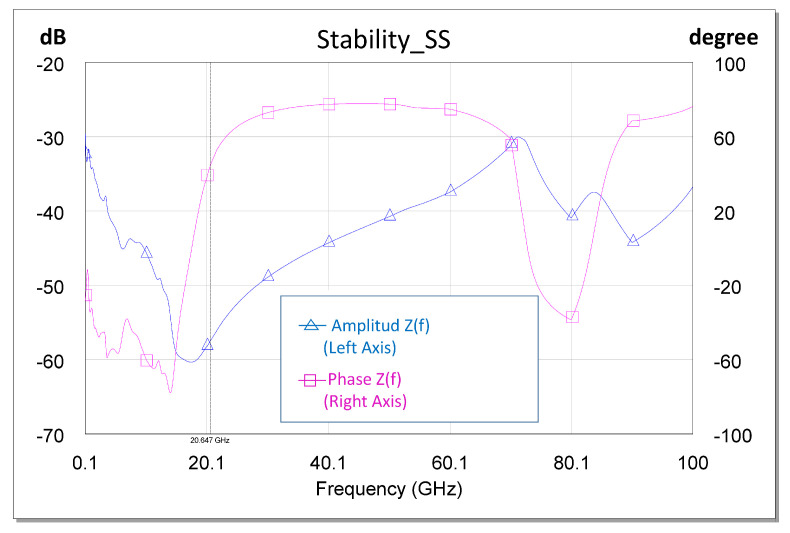
Small-signal stability analysis.

Based on pure observation, pole-zero extraction techniques such as those presented in [18,19,21] can be used. We can surmise that only unstable poles can initiate an oscillation at a given frequency if the phase conditions are appropriate. To ensure the existence of an unstable pole in an impedance function (*Z*(*f*)), we have to ensure the existence of the pole and that the phase at said pole is increasing. The reasoning is analogous when we use the Y(f) function. Separating the real (*Real($Z(f_{o})$)*) and imaginary (*Imaginary($Z(f_{o})$)*) parts of the function, it must be true that *Real($Z(f_{o})$) < 0* and *Imaginary($Z(f_{o})$) = 0*, with the condition that *d(Imaginary($Z(f_{o}$)))/df > 0*. In the opinion of the authors, it is easier to observe the poles physically and verify that the phase is not increasing. Figure 15 shows the small-signal stability analysis for the X HPA, where the stability of the designed circuit can be checked.

### 3.2. Large-Signal Stability Analysis

**Figure 16:**
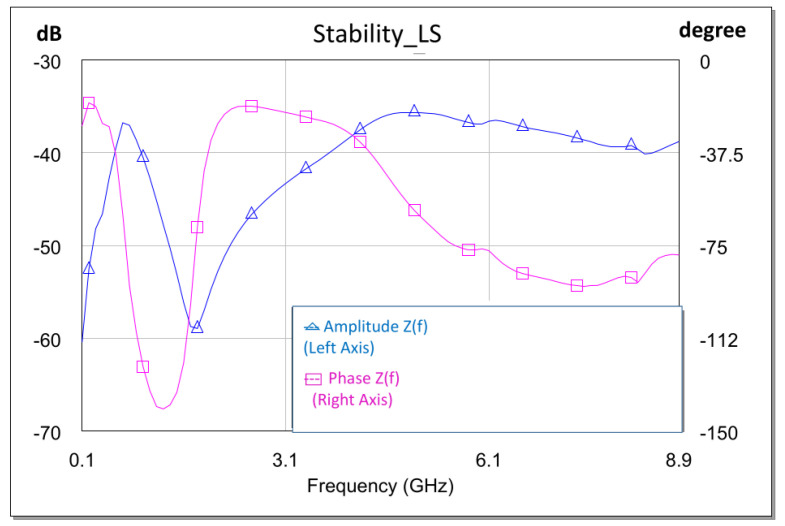
Large-signal stability analysis: $P_{in}$ = 16 dBm.

The large-signal stability analysis was performed at the design level, selecting nodes that were considered sensitive based on clues from the small-signal analysis. The large-signal test bench was similar to the small-signal test bench, with the exception that the analysis was performed in the presence of a large signal. Therefore, we generated a graph for each power and can analyze its evolution as a function of the power. As in the previous case, we only consider the poles to verify that they do not have an increasing phase, since this, as in the previous case, would indicate unstable poles (see Figure 16).

The analysis was divided into two steps. In the first step, a wide frequency interval was used (broadband), with the objective of detecting the frequencies of possible compliance with the oscillation start conditions of the function evaluated in the particular circuit node. In cases that present doubts, a narrow frequency interval around these points was analyzed to achieve greater precision in the determination of possible instabilities.

As a second step, the process was repeated for different power levels at the HPA input. This allowed us to see the evolution of the stability as a function of the excitation level at the input of the X HPA. Figure 16 shows the fulfillment of the stability conditions, with a power at the input of the X HPA of $P_{in}$ = 16 dBm.

At this point, taking into account the design spiral, if we have enough margin, we can decrease the resistive padding (mentioned in Section 3.1) and, thus, improve the gain performance of the design. These steps were performed in previous stages of the design.

## 4. Measurement Problems

Below, we describe the problems that occurred and that we think are of interest to MMIC designers. After verifying the stability of the circuit for these operating conditions, we proceeded to the “on-wafer” measurement of the circuit. This measurement could not be performed in the first instance because the circuit presented a small oscillation in the *VHF* band (≈40 MHz). With these data, we proceeded to the analysis of the “on-wafer” measurement test that was set up for this purpose.

This point is important for designers because the design work is often carried out isolation from the engineers in charge of carrying out the measurements. Therefore, justifying and showing the validity of the design process helps to find faults and prevent errors in future designs. In the HPA-MMIC design presented in this paper, the failures were due, first, to the lack of precision in the manufacturing process of the plates used to measure the chip and, secondly, to the failure to glue the chip to the mass of the carrier for measurement.

**Figure 17:**
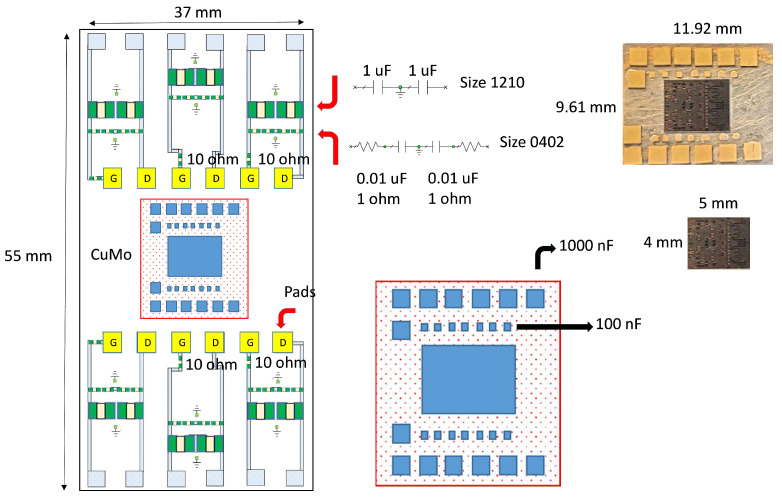
Schematic representation of the MMIC in the carrier. The MMIC is mounted on a carrier where the capacitors are located according to the figure, in the lower right part. Small blue rectangles represent 100 nF capacitors, and larger blue boxes represent 1000 nF capacitors. All this with bondings between stages. The size of this carrier is 11.92 × 9.61 mm. At the top right, there is a photograph of it. Furthermore, this carrier is mounted on a printed circuit board (PCB) which is responsible for powering the circuit. The size of this PCB is 37 × 52 mm. Additionally, the biases of the different stages are located on the PCB. The yellow boxes are the smallest pads in the photo. Bondings are mounted on these pads that go to the HPA MMIC carrier. The G indicate the gates and the D the drains of the different stages. Finally, the arrows indicate the schematic and how the 1 uF decoupling capacitors, size (1210), and the 0.01 uF SCR capacitors, size (0402) have been assembled.

**Figure 18:**
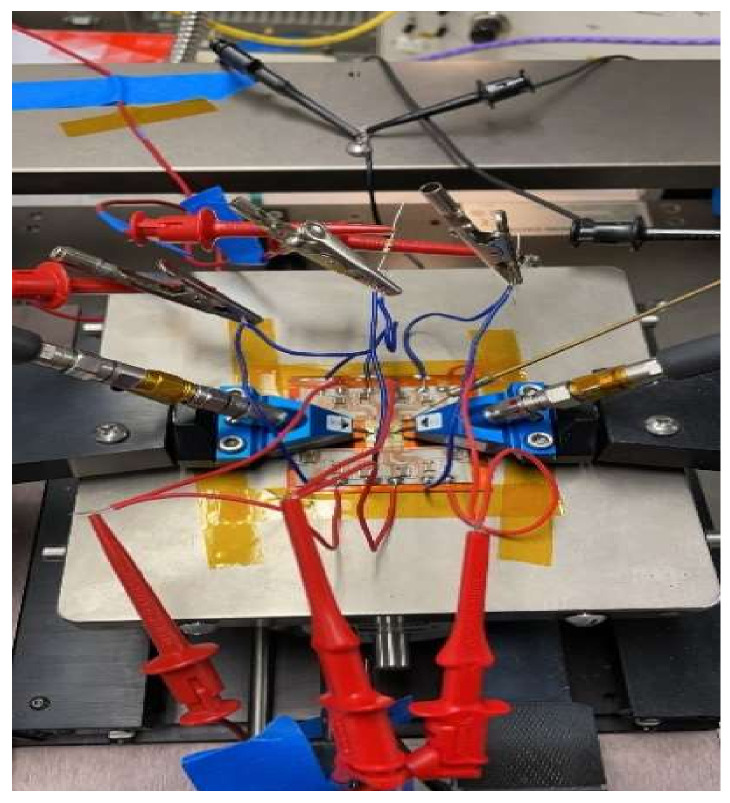
Photograph of MMIC carrier measurement.

Since the coplanar probes of the input and output signals did not have sufficient induction and having seen the photographs of the assembly, where there was a significant path and induction caused by the feeding probes attached to the sources, we proceeded to the assembly of the circuit, as shown in Figure 17, with the help of a carrier. The assembly was obtained with the MMIC shown in Figure 18.

**Figure 19:**
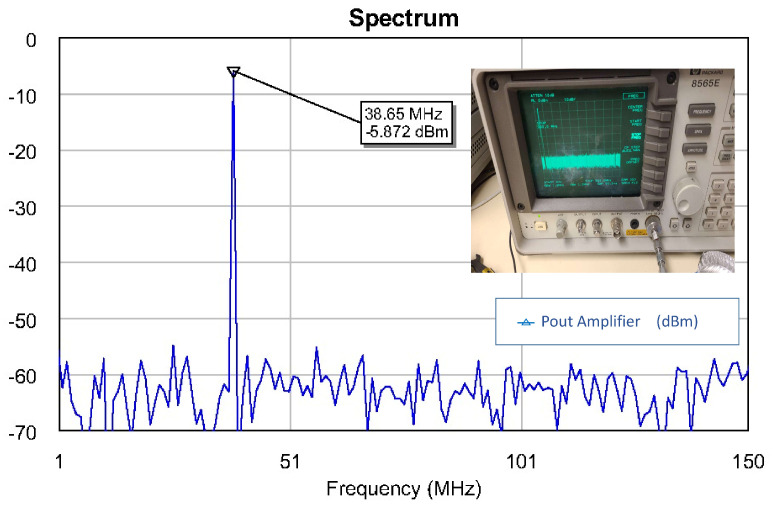
Oscillation in the MMIC.

As in the previous case, it could not be measured at first. In addition, it presented a low-frequency oscillation (≈40 MHz) that had not been detected in any of the stability simulations, as shown in Figure 19.

The circuit was measured and mounted on the carrier circuit, the influence of the different sections of the HPA circuit on the detected oscillation was investigated, and the source of circuit instability was identified.

**Figure 20:**
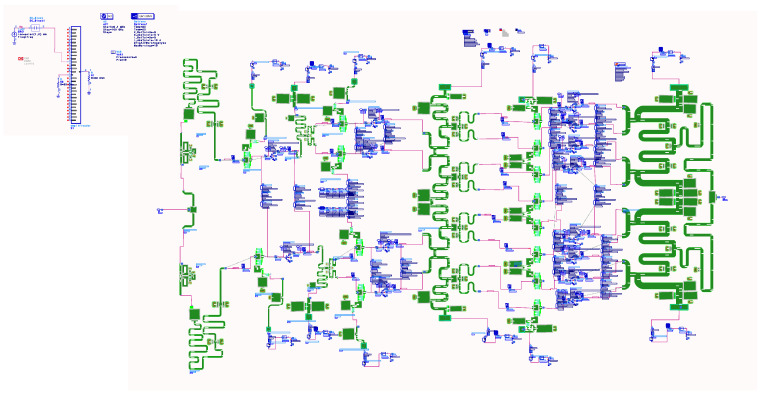
ADS setup: small-signal stability analysis with parasite inductance.

To identify the instability in the simulation and in order to introduce external elements that could induce instabilities in the circuit being simulated, the networks were divided schematically between the different stages, as shown in the ADS [31] of Figure 20.

**Figure 21:**
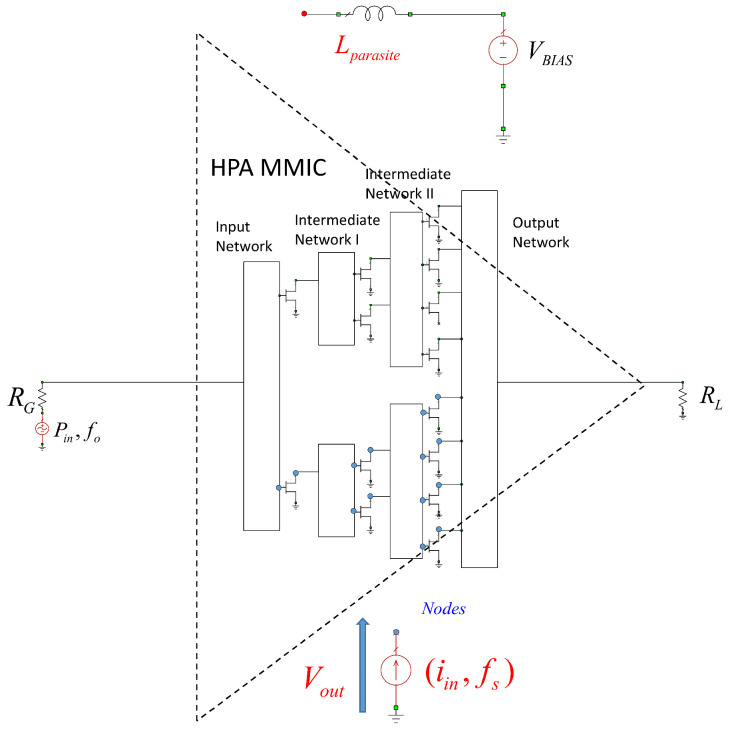
Scheme of the analysis of Z(f) in the large signal, looking for instability with $L_{parasite}$ and pole-zero identification.

We proceeded to analyze the circuit, and, as in the previous case, we performed an analysis of the pole-zero stability. Figure 21 shows the simplified scheme of the small-signal stability analysis with a parasitic inductance. As in the previous case, to show the complexity of the analysis, we thought that it would be convenient to show the simulation scheme carried out in the ADS simulator [31]. Stability analysis was performed by placing a parasitic inductance in the power supplies, as shown in the ADS scheme in Figure 20. ADS was used both to corroborate the AWR [32] analysis and as a redundancy check.

**Figure 22:**
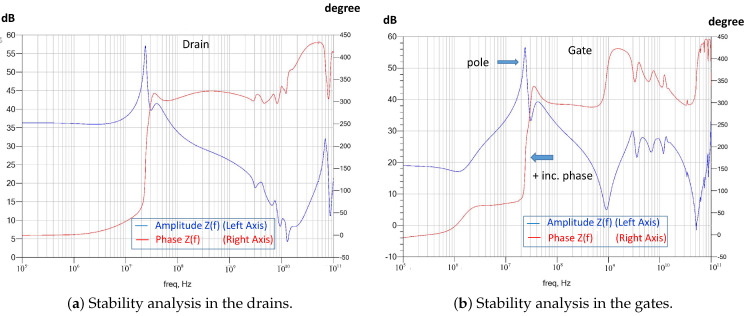
Stability analysis with $L_{parasite}$ = 1200 nH. As there is an increase in phase, with the red line we look for this increase to correspond to the position of a pole.

The evolution of the pole-zero stability was analyzed versus the value of inductance, L. The solution showed instabilities (see Figure 22). There were unstable poles due to high values of parasitic inductance. The measured circuit, both on wafer and mounted on the carrier, was found to be fed by probes with a high parasitic inductance. Furthermore, it was observed that the carrier had contacted the dough incorrectly because the conductive putty did not make sufficient contact. Thus, all these defects were modeled using parasitic inductance, and the simulation was carried out. Figure 22 shows the stability analysis considering amplifier biases and a mass return with a value of $L_{parasite}$ = 1200 nH. In the figure, the location of the unstable pole is indicated by two arrows. Here, the positive slope tells us that there is an unstable pole at a frequency close to the oscillation.

**Figure 23:**
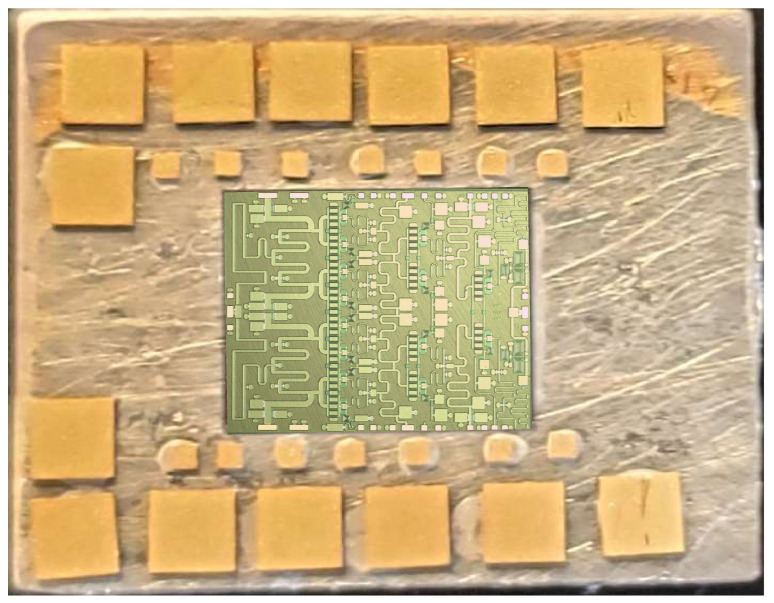
Detailed view of the MMIC glued on the carrier.

In addition, it was found that instability appeared to occur at values lower than $L_{parasite}$ = 400 nH. With the aid of the microscope shown in Figure 23, we also observed that the epoxy adhesive with which the MMIC had been fixed had undergone a defective drying by measuring its continuity with the ground. Additionally, it was observed that this value was ≈20 Ω, which caused the instability.

**Figure 24:**
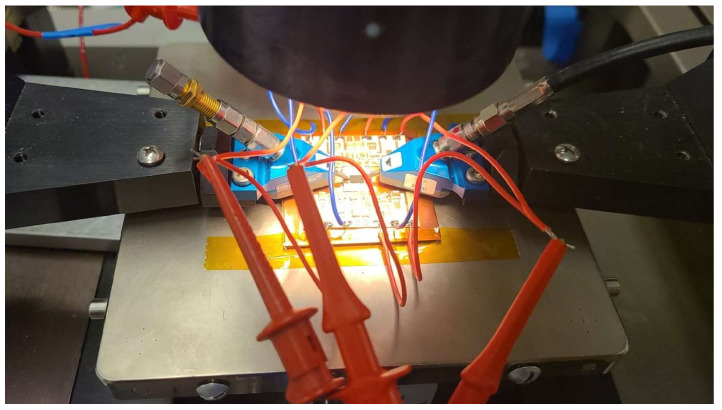
Device parameter measurements.

The test bench was reassembled to measure the circuit in a totally stable state as predicted by the analyses. The behavior of the amplifier was perfectly measured, as shown in Figure 24. The final measurements are shown in the next section.

## 5. Final Measurements

**Figure 25:**
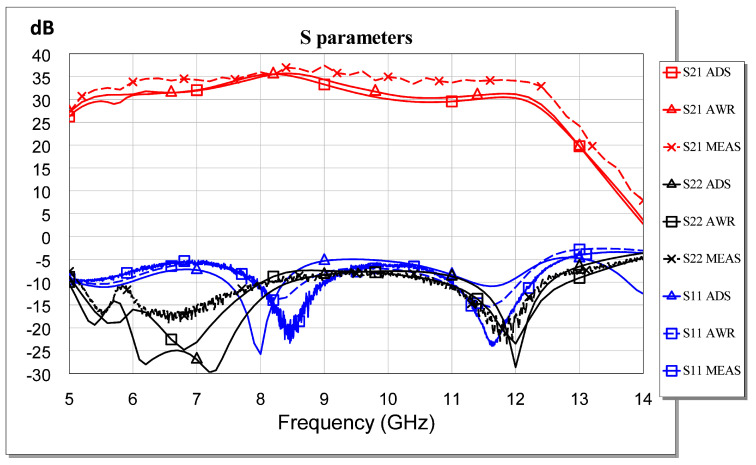
Measured vs. simulated S parameters.

**Figure 26:**
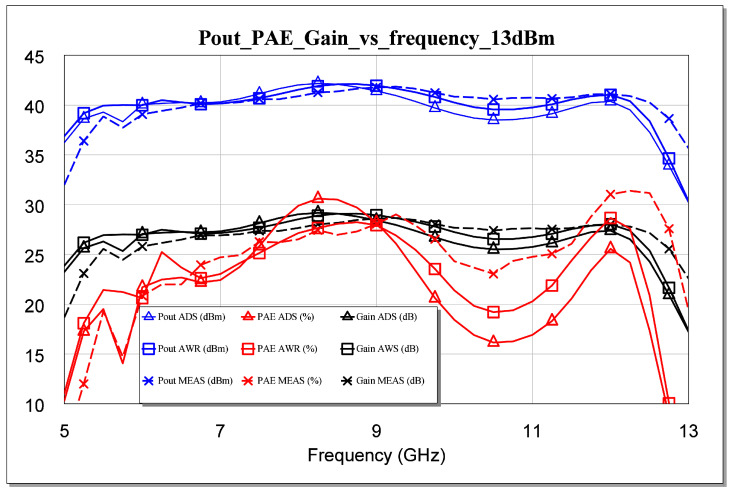
Measured $P_{out}$, gain, and PAE parameters versus simulated frequency for $P_{in}$ = 13 dBm.

**Figure 27:**
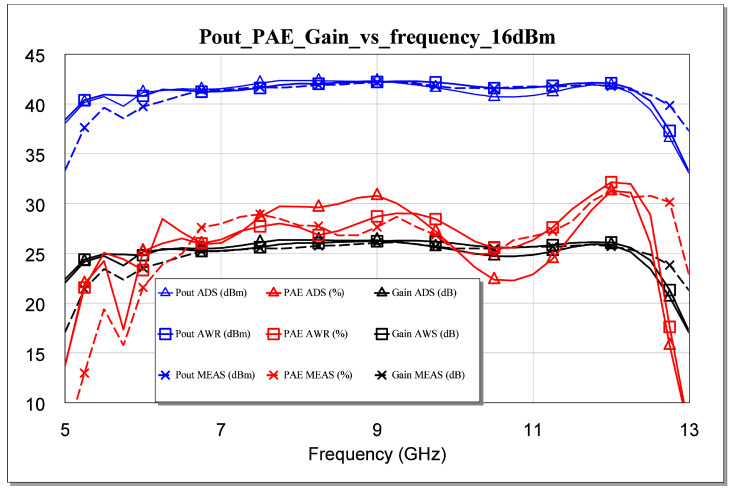
Measured $P_{out}$, gain, and PAE parameters versus simulated frequency for $P_{in}$ = 16 dBm.

**Figure 28:**
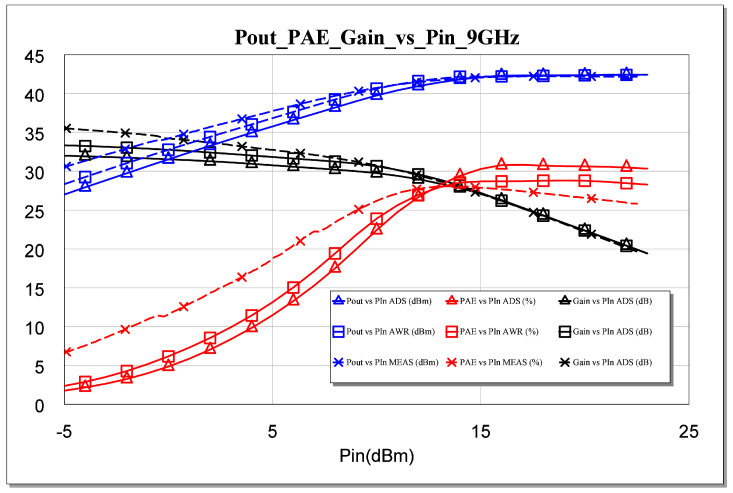
Measured $P_{out}$, gain, and PAE parameters versus simulated $P_{in}$ for *f* = 9 GHz.

**Figure 29:**
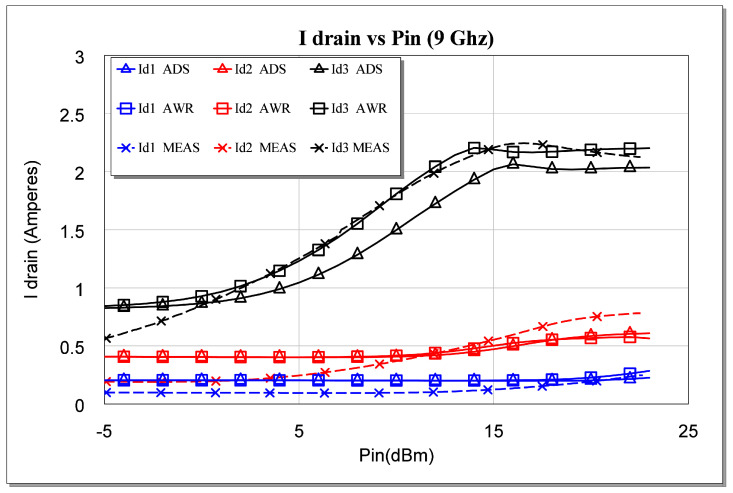
Measured $I_{d1}$, $I_{d2}$, and $I_{d3}$ parameters versus simulated $P_{in}$ for *f* = 9 GHz.

The failure of our first attempts to measure the MMIC occurred not because of its instability but because of errors or defects in the measurement. Therefore, we solved the above issue and carried out the measurements. As a result, an excellent correlation between the measurement and the simulation was observed. All measurements are shown in Figure 25, Figure 26, Figure 27, Figure 28 and Figure 29. Specifically, the result obtained from the measurements carried out once the instability identified in the previous sections was corrected is shown. We also show a comparison of the measurement and simulations results obtained with both AWR version 16 and ADS 2023 design softwares.

Figure 25 shows the excellent agreement between the ADS-AWR simulations and the *S*-parameter measurement results. The maximum value of the small-signal parameter ($S_{21}$) is near 35 dB, and the input return loss ($S_{11}$) is greater than 5 dB. In addition, the output return loss ($S_{22}$) is greater than 8 dB.

The measurements for two powers carried out on the large signal are presented below. For $P_{in}$ = 13 dBm, our output power was higher than 40 dBm, with a gain of 27 dB and a maximum PAE of 32%, as shown in Figure 26. For 16 dBm, our $P_{out}$ was higher than 42 dBm, with a gain greater than 25 dB and a maximum PAE of 34%, as shown in Figure 27. The results corroborate the excellent correlation between the simulations and the measurements.

If a compression analysis were performed for 9 GHz, as shown in Figure 28, we would expect to see that this process presents a smooth compression, unlike GaAs, which allows it to work with powers much higher than the 1 dB compression point.

Finally, as shown in Figure 29, we can also analyze the behavior of the currents of the three stages as a function of frequency for the sizing of our power supplies. The abovementioned analysis was also performed at 9 GHz as an example of the excellent correlation between the simulations and the measurements.

## 6. Discussion

The obtained results demonstrate that the performance of the proposed HPA is satisfactory compared with the performance of other HPAs reported in the literature on this topic. Table 1 shows a comparison of our proposal with six very good proposals that can be considered references in the field of HPA design.

In Table 1, $V_{D}$ is the drain bias voltage; $f_{lower}-f_{upper}$ are the lower ($f_{lower}$) and upper ($f_{upper}$) cutoff frequencies of the bandwidth (BW (13)), respectively; $f_{o}$ is the center frequency (14); $\Delta f_{o}$ is the percentage bandwidth (15); $P_{out}$ is the output power; PAE is the power-added efficiency; CW stands for continuous wave; NA stands for not applicable; and Time/Duty is the pulse width of the modulating or pulsed signal with respect to one duty cycle.

(13) $$\begin{array}{c} BW=f_{upper}-f_{lower} \end{array}$$

(14) $$\begin{array}{c} f_{o}=\frac{f_{upper}\;+\;f_{lower}}{2} \end{array}$$

(15) $$\begin{array}{c} \Delta f_{o}=\frac{BW}{f_{o}}\cdot 100 \end{array}$$

This table demonstrates that our design is in accordance with the current state of the art of HPAs. It is worth highlighting that the bandwidth of our HPA ranges from 5.3 GHz to 12.3 GHz. This is the HPA with the highest bandwidth among all those compared in the table. In addition, our amplifier presents good performance between 5.2 GHz and 12.8 GHz, with an output power greater than 8 W (39 dBm).

The motivation of our paper has a similar origin to the case reported in [9]. In [9], several of the designs oscillated when measured “on wafer”. Likewise, in our research, our design also oscillated when the first “on-jig” measurements were carried out. Therefore, in our paper, we justified the reason for the oscillation of our HPA. Furthermore, we reflected on the stability methods, verified that the HPA was well designed, and discovered that the oscillation was due to a mounting defect of the MMIC on the jig.

Finally, it is worth mentioning that in our case, the study of harmonics is not appropriate because the MMIC design presented in this research has a wide bandwidth of greater than one octave. Therefore, a compromise was established between the PAE value and the bandwidth. In short, it is not possible to obtain very large PAE values working with very large bandwidths. In other words, to obtain large PAE values, the designer has to work with small bandwidths, as is the case of the research presented in [33,34], in which the authors worked with reduced bandwidths.

**Table 1:** Comparative analysis of the performance of different HPAs.

HPA	Technology	$V_{D}$ (V)	$f_{\mathrm{lower}}-f_{\mathrm{upper}}$ (GHz)	$\Delta f_{o}$ (%)	Operation	Stages	Time/Duty (μs/%)	$P_{\mathrm{out}}$ (W)	Gain (dB)	PAE (%)
This	0.15 μm	20	5.3–12.3	100	AB	3	100/10	8–16	25–28	38–43
Work	GaN									
[35]	0.25 μm	25	9–11	20	AB	2	20/10	35–45	19	40–52
	GaN									
[36]	0.25 μm	28	7.8–8.8	12	AB	2	CW	22–22.5	26	50
	GaN									
[33]	0.15 μm	30	8.2–8.9	8	AB	1	NA/10	28–31.5	11–12	57–60
	GaN									
[34]	0.15 μm	44	9.8–11.5	8	CascodeF	1	NA/10	5.8–9.2	7–12	50–62
	GaN									
[37]	0.25 μm	28	8–12	40	AB	3	100/10	56.2–74	21–26	40–45
	GaN									
[38]	0.15 μm	20	8–12.5	44	AB	2	25/10	14–17	23	50–62
	GaN									

## 7. Conclusions

This paper describes the design of a high-power GaN MMIC amplifier with a small-signal gain of at least 32 dB, 32.8% peak power-added efficiency, and saturated output power of over 42.8 dBm from 5.3 to 12.3 GHz at a drain bias voltage of 20 V. Herein, we described the design process in detail and how oscillations occurred in the “on-wafer” measurement and in the measurement of the chip mounted on the “carrier circuit”. Additionally, errors that led to instabilities were identified.

In this research, an in-depth analysis of the root causes of instabilities was performed early in the HPA-MMIC development process, and this knowledge was used in all aspects of the final design. This highlighted the need for a robust stability diagnostic tool to identify the loops through which feedback occurs. Simulation tools were used to identify instabilities produced by incorrect mounting on the carrier or by inadequate decoupling of the bias probes.

## Data Availability

Data are contained within the article.

## References

[1] Miranda R.F., Barriquello C.H., Reguera V.A., Denardin G.W., Thomas D.H., Loose F., Amaral L.S. (2023). Review of Cognitive Hybrid Radio Frequency/Visible Light Communication Systems for Wireless Sensor Networks. Sensors.

[2] Abdulwali Z.S.A., Alqahtani A.H., Aladadi Y.T., Alkanhal M.A.S., Al-Moliki Y.M., Aljaloud K., Alresheedi M.T. (2023). A High-Performance Circularly Polarized and Harmonic Rejection Rectenna for Electromagnetic Energy Harvesting. Sensors.

[3] Eidaks J., Kusnins R., Babajans R., Cirjulina D., Semenjako J., Litvinenko A. (2023). Efficient Multi-Hop Wireless Power Transfer for the Indoor Environment. Sensors.

[4] Costanzo A., Augello E., Battistini G., Benassi F., Masotti D., Paolini G. (2023). Microwave Devices for Wearable Sensors and IoT. Sensors.

[5] Kouhalvandi L., Matekovits L., Peter I. (2023). Amplifiers in Biomedical Engineering: A Review from Application Perspectives. Sensors.

[6] Van Der Bent G., De Hek A.P., Van Vliet F.E., Ouarch Z. Single-Chip 100-Watt S-band Power Amplifier in 0.25 μm GaN HEMT MMIC Technology. Proceedings of the 15th European Microwave Integrated Circuits Conference, EuMIC2020.

[7] Qorvo QPA3069 Data Sheet. https://www.qorvo.com/products/p/QPA3069.

[8] Kwiatkowski P., Knioła M., Szczepaniak Z. (2023). Microwave Frequency Doubler with Improved Stabilization of Output Power. Sensors.

[9] Platzker A., Struble W., Hetzler K.T. Instabilities diagnosis and the role of k in microwave circuits. Proceedings of the 1993 IEEE MTT-S International Microwave Symposium Digest.

[10] Struble W., Platzker A. A rigorous yet simple method for determining stability of linear N-port networks [and MMIC application]. Proceedings of the 15th Annual GaAs IC Symposium.

[11] Woods D. (1976). Reappraisal of the unconditional stability criteria for active 2-port networks in terms of s parameters. IEEE Trans. Circuits Syst..

[12] Edwards M.L., Sinsky J.H. (1992). A new criterion for linear 2-port stability using a single geometrically derived parameter. IEEE Trans. Microw. Theory Tech..

[13] Jiménez-Martín J.L., Gonzalez-Posadas V., Parra-Cerrada Á., Blanco-del-Campo A., Segovia-Vargas D. (2012). Transpose Return Relation Method for Designing Low Noise Oscillators. Prog. Electromagn. Res. (PIER).

[14] Jiménez-Martín J.L., Gonzalez-Posadas V., Parra-Cerrada Á., Segovia-Vargas D., Garcia-Munoz L.E. (2012). Provisos for Classic Linear Oscillator Design Methods. New Linear Oscillator Design Based on the NDF/RRT. Prog. Electromagn. Res. (PIER).

[15] Freitag R.G. A unified analysis of mmic power amplifier stability. Proceedings of the 1992 IEEE MTT-S Microwave Symposium Digest.

[16] Wang K., Jones M., Nelson S. The S-probe-a new, cost-effective, 4-gamma method for evaluating multi-stage amplifier stabilityy. Proceedings of the 1992 IEEE MTT-S Microwave Symposium Digest.

[17] Narhi T., Valtonen M. Stability envelope-new tool for generalised stability analysis. Proceedings of the 1997 IEEE MTT-S International Microwave Symposium Digest.

[18] Ayllon N., Collantes J., Anakabe A., Lizarraga I., Soubercaze-Pun G., Forestier S. (2011). A Systematic Approach to the Stabilization of Multitransistor Circuits. IEEE Trans. Microw. Theory Tech..

[19] Anakabe A., Mons S., Gasseling T., Casas F., Quere R., Collantes J.M., Malet A.A. Efficient nonlinear stability analysis of microwave circuits using commercially available tools. Proceedings of the European Microwave Week Conference.

[20] Mons S., Nallatamby J.-C., Quere R., Savary P., Obregon J. (1999). A unified approach for the linear and nonlinear stability analysis of microwave circuits using commercially available tools. IEEE Trans. Microw. Theory Tech..

[21] Hernandez S., Suarez A. (2019). Systematic methodology for the global stability analysis of nonlinear circuits. IEEE Trans. Microw. Theory Tech..

[22] AMCAD Engineering STAN Tool: A Unique Solution for the Stability Analysis of RF & Microwave Circuits. https://www.amcad-engineering.com/content/uploads/2018/06/Application_Note_STAN.pdf.

[23] WIN Semiconductors Home Page. https://www.winfoundry.com/en-US.

[24] Pino J.d., Khemchandani S.L., Mayor-Duarte D., San-Miguel-Montesdeoca M., Mateos-Angulo S., de Arriba F., García M. (2023). A Ku-Band GaN-on-Si MMIC Power Amplifier with an Asymmetrical Output Combiner. Sensors.

[25] Fano R.M. (1948). Theoretical limitations on the broadband matching of arbitrary impedances. J. Frankl. Inst..

[26] Lee H., Park H.-G., Le V.-D., Nguyen V.-P., Song J.-M., Lee B.-H., Park J.-D. (2023). X-band MMICs for a Low-Cost Radar Transmit/Receive Module in 250 nm GaN HEMT Technology. Sensors.

[27] Galante-Sempere D., Khemchandani S.L., del Pino J. (2023). A 2-V 1.4-dB NF GaAs MMIC LNA for K-Band Applications. Sensors.

[28] Kouhalvandi L. (2022). Directly Matching an MMIC Amplifier Integrated with MIMO Antenna through DNNs for Future Networks. Sensors.

[29] Niclas W.T., Wilser R.R., Gold R.B., Hitchens W.R. (1980). The matched feedback amplifier: Ultrawide-band microwave amplification with GaAs MESFET’s. IEEE Trans. Microw. Theory Tech..

[30] Rollet J.M. (1962). Stability and power-gain invariants of linear two ports. IRE Trans. Circuit Theory.

[31] Keysight PathWave Advanced Design System (ADS). https://www.keysight.com/us/en/products/software/pathwave-design-software/pathwave-advanced-design-system.html.

[32] Cadence R.F. Microwave Design with AWR Software. https://www.cadence.com/en_US/home/tools/system-analysis/rf-microwave-design.html.

[33] Kawamura Y., Hangai M., Mizutani T., Tomiyama K., Yamanaka K. 30W output/60% PAE GaN power amplifier at X-band 8% relative bandwidth. Proceedings of the 8th 2016 Asia-Pacific Microwave Conference (APMC).

[34] Kang J., Moon J.-S. (2017). Highly efficient wideband X-band MMIC class-F power amplifier with cascode FP GaN HEMT. Electron. Lett..

[35] Piotrowicz S., Ouarch Z., Chartier E., Aubry R., Callet G., Floriot D., Jacquet J.C., Jardel O., Morvan E., Reveyrand T. 43W, 52% PAE X-Band AlGaN/GaN HEMTs MMIC amplifiers. Proceedings of the 2010 IEEE MTT-S International Microwave Symposium Digest.

[36] Couturier A.M., Poitrenaud N., Serru V., Dionisio R., Fontecavc J.J., Camiade M. 50 % High Efficiency X-Band GaN MMIC Amplifier for Space Applications. Proceedings of the 48th European Microwave Conference (EuMC).

[37] Tao H.-Q., Hong W., Zhang B., Yu X.-M. (2017). A Compact 60W X-Band GaN HEMT Power Amplifier MMIC. IEEE Microw. Wirel. Components Lett..

[38] Ayad M., Poitrenaud N., Serru V., Camiade M., Gruenenpuett J., Riepe K.J. A High Efficiency MMIC X-band GaN Power Amplifier. Proceedings of the 50th European Microwave Conference (EuMC).

